# Ribosomopathies: how a common root can cause a tree of pathologies

**DOI:** 10.1242/dmm.020529

**Published:** 2015-09-01

**Authors:** Nadia Danilova, Hanna T. Gazda

**Affiliations:** 1 Department of Molecular, Cell & Developmental Biology, University of California, Los Angeles, CA 90095, USA; 2 Division of Genetics and Genomics, The Manton Center for Orphan Disease Research, Boston Children's Hospital, Boston, MA 02115, USA; 3 Department of Pediatrics, Harvard Medical School, Boston, MA 02115, USA; 4 Broad Institute, Cambridge, MA 02142, USA

**Keywords:** Ribosome biogenesis, Ribosomal protein, Ribosomopathy, Diamond-Blackfan anemia, p53, *ΔNp63*

## Abstract

Defects in ribosome biogenesis are associated with a group of diseases called the ribosomopathies, of which Diamond-Blackfan anemia (DBA) is the most studied. Ribosomes are composed of ribosomal proteins (RPs) and ribosomal RNA (rRNA). RPs and multiple other factors are necessary for the processing of pre-rRNA, the assembly of ribosomal subunits, their export to the cytoplasm and for the final assembly of subunits into a ribosome. Haploinsufficiency of certain RPs causes DBA, whereas mutations in other factors cause various other ribosomopathies. Despite the general nature of their underlying defects, the clinical manifestations of ribosomopathies differ. In DBA, for example, red blood cell pathology is especially evident. In addition, individuals with DBA often have malformations of limbs, the face and various organs, and also have an increased risk of cancer. Common features shared among human DBA and animal models have emerged, such as small body size, eye defects, duplication or overgrowth of ectoderm-derived structures, and hematopoietic defects. Phenotypes of ribosomopathies are mediated both by p53-dependent and -independent pathways. The current challenge is to identify differences in response to ribosomal stress that lead to specific tissue defects in various ribosomopathies. Here, we review recent findings in this field, with a particular focus on animal models, and discuss how, in some cases, the different phenotypes of ribosomopathies might arise from differences in the spatiotemporal expression of the affected genes.

## Abstract (precis)

**Summary:** This paper reviews recent data on Diamond Blackfan anemia and discusses them in connection with other ribosomopathies.

## Introduction

Diamond-Blackfan anemia (DBA) is a congenital syndrome associated with anemia, physical malformations and cancer (Halperin and Freedman, 1989; Vlachos et al., 2012). In the majority of individuals with DBA, mutations or gene deletions of a subset of ribosomal proteins (RPs; see Box 1) are found, with *RPS19* mutations accounting for about 25% of all cases (Table 1). Although some mutations are dominant negative, the major mechanism of the disease is associated with haploinsufficiency of an RP that disrupts the processing of pre-ribosomal RNA (pre-rRNA), leading to abortive ribosome biogenesis (Choesmel et al., 2007; Devlin et al., 2010; Farrar et al., 2014; Flygare et al., 2007; Gazda et al., 2004; Leger-Silvestre et al., 2005; Panic et al., 2006).

**Box 1. Glossary**

**5′ and 3′ external transcribed spacers (ETSs):** non-functional RNA sequences of the pre-rRNA transcript that have structural roles and are excised during pre-rRNA processing.

**5q-myelodysplastic syndrome (5q-MDS):** a form of MDS that is caused by loss of a part of the q arm of chromosome 5.

**Adenosine deaminase (ADA):** an enzyme involved in the metabolism of adenosine.

**Anemia:** a condition associated with an insufficient number of red blood cells in blood.

**Asplenia:** absence of spleen or very small spleen.

**Biliary cirrhosis:** cirrhosis caused by damage to the bile ducts in the liver.

**Epiboly:** growth of a cell layer to envelope the yolk during gastrulation.

**Haploinsufficiency:** when a single copy of the gene is insufficient to maintain normal function.

**Internal ribosome entry site (IRES):** a sequence inside mRNA that allows for initiation of cap-independent translation in the middle of mRNA.

**Internally transcribed spacers (ITSs):** spacers between 18S, 5.8S and 28S rRNA in the pre-rRNA transcript that play structural roles and, like ETSs, are excised during pre-rRNA processing.

**Jaundice:** yellow color of the skin and whites of the eyes caused by excess bilirubin in the blood.

**Macrocytic erythrocytes:** abnormally large red blood cells.

**Mechanistic target of rapamycin (mTOR):** a serine/threonine kinase that is a central regulator of cellular metabolism. It forms mTORC1 and mTORC2 complexes, which mediate cellular responses to stresses such as DNA damage and nutrient deprivation.

**Myelodysplastic syndrome (MDS):** a syndrome caused by mutations in several genes, most often encoding splicing factors. It is associated with ineffective production of blood, which often leads to leukemia.

**Reticulocyte:** immature erythrocyte.

**RNA polymerase I and III (PolI/PolIII):** enzymes involved in the transcription of non-coding RNAs.

**Ribosomal proteins (RPs):** proteins that together with ribosomal RNA (rRNA) make up the ribosome. They are called RPS (RP from small ribosomal subunit) or RPL (RP from large ribosomal subunit) depending on whether they associate with the small or large subunit of the ribosome.

**Small nucleolar RNA (snoRNA):** a class of small RNAs that guide chemical modifications such as methylation and pseudouridylation of other RNAs.

**Table 1.**
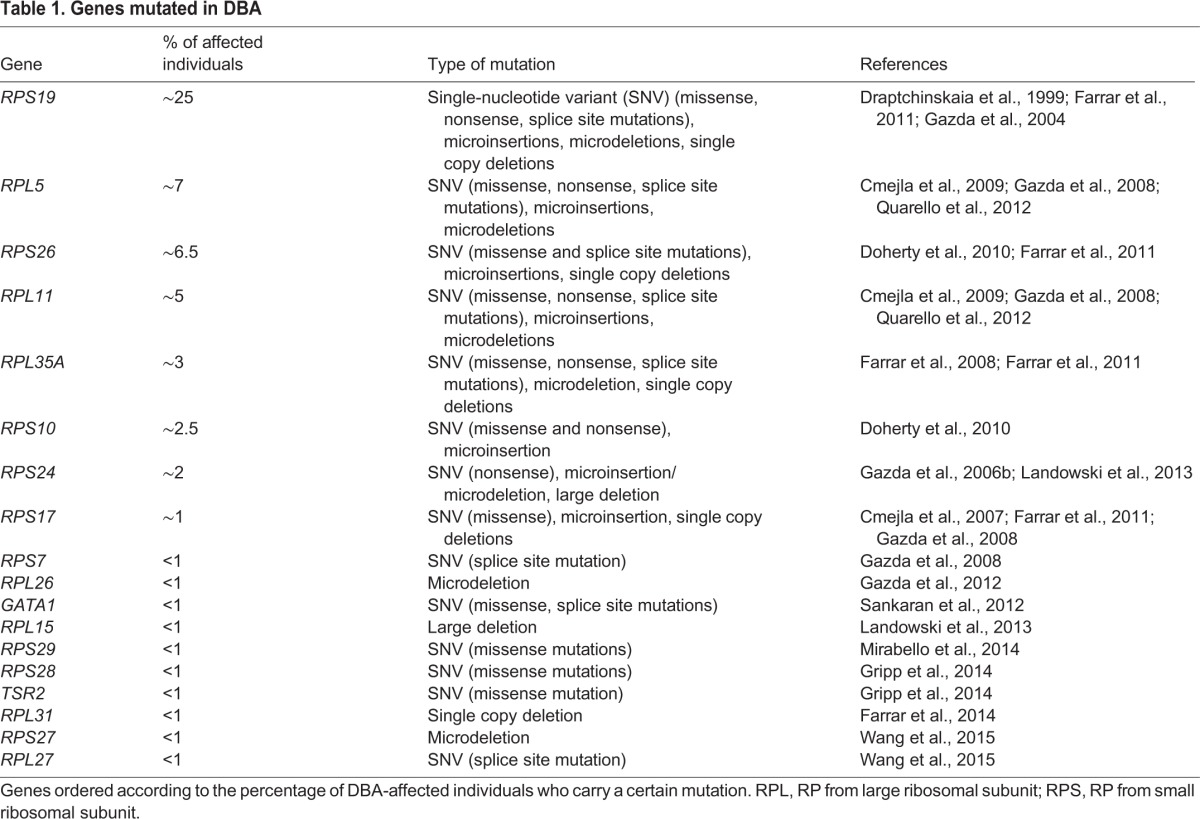
**Genes mutated in DBA**

DBA is a rare disease with an incidence of ∼5 cases per million live births, but it has attracted substantial attention as a model disease for ribosomopathies, a group of pathologies associated with defects in ribosome biogenesis (Armistead and Triggs-Raine, 2014; James et al., 2014). Despite this common defect, phenotypes of ribosomopathies differ. A common feature among several ribosomopathies is p53 activation (Danilova et al., 2008b; Elghetany and Alter, 2002; Jones et al., 2008), but the mechanisms involved have not been completely elucidated. A p53-independent response to RP deficiency has also been observed (Aspesi et al., 2014; Danilova et al., 2008b; Singh et al., 2014; Torihara et al., 2011). The pathways that lead from a particular defect in ribosome biogenesis to the phenotype of a ribosomopathy are still not well understood. Several insightful reviews about the mechanisms of ribosomopathies have been published recently (Armistead and Triggs-Raine, 2014; Ellis, 2014; Golomb et al., 2014; James et al., 2014; Ruggero and Shimamura, 2014) with a focus on human data. However, cross-species analysis might also provide additional clues as to the mechanisms of these diseases.

Here, we review the phenotypes that are caused by RP deficiency in humans as well as in mouse, fly and zebrafish models, with an emphasis on their common features. We also discuss the consequences of ribosomal stress at the molecular level, with a particular emphasis on p53 activation, metabolic changes, the origin of erythroid defects, and congenital malformations. We also discuss potential new directions for DBA treatment that arise from recent findings.

## Phenotypes caused by defects in ribosome biogenesis

All ribosomopathies originate from defects in ribosome biogenesis, yet every ribosomopathy has a unique phenotype with different tissues affected. In this section, we provide an overview of the phenotypes caused by RP deficiency in human DBA and in its animal models, and compare them to phenotypes of other ribosomopathies.

### RP deficiency

In humans, DBA is often diagnosed during the first year of life; common clinical features include anemia, low reticulocyte count, macrocytic erythrocytes (see Box 1 for a glossary of terms), increased expression of fetal hemoglobin and elevated activity of adenosine deaminase (ADA; Box 1) (Fargo et al., 2013; Halperin and Freedman, 1989). Approximately 40% of affected individuals have short stature and variable congenital malformations of craniofacial skeleton, eyes, heart, visceral organs and limbs. Notably, duplication of some structures, including triphalangeal thumb, bifid thumb and extra ribs, has been reported (Halperin and Freedman, 1989). The clinical symptoms of DBA rarely correlate with the type of causative mutation. However, individuals with mutations in *RPL5* often have a cleft palate, whereas this malformation was not observed in individuals carrying *RPS19* mutations (Gazda et al., 2008). The acquired haploinsufficiency of *RPS14* has been shown to underlie the erythroid defect in 5q-myelodysplastic syndrome (MDS) (Ebert et al., 2008; Pellagatti et al., 2008).

Individuals with DBA have an increased frequency of both solid cancers and leukemia (Vlachos et al., 2012), and acquired mutations in *RPL5* and *RPL10* are found in T-cell acute lymphoblastic leukemia (De Keersmaecker et al., 2013).

A strikingly different phenotype is associated with the RPSA protein, an RP that is involved in the maturation of the 40S subunit and also serves as a laminin receptor. Mutations in this RP lead to familial isolated congenital asplenia (see Box 1 for a glossary of terms) (Bolze et al., 2013). However, this phenotype is likely associated with the function of RPSA as a laminin receptor because laminin deficiency has been shown to lead to asplenia (Miner and Li, 2000).

In the fruit fly, *Drosophila*, the haploinsufficiency of many RPs leads to a Minute phenotype, which is characterized by developmental delay, small size, small bristles, small rough eyes, and recessive lethality at late embryonic/early larval stages (Kongsuwan et al., 1985; Marygold et al., 2005). Notably, heterozygous *Rpl38* and *Rpl5 Drosophila* mutants have abnormally large wings (Marygold et al., 2005).

Zebrafish embryos homozygous for RP mutations also exhibit developmental delay and small size as well as a small head and eyes, brain apoptosis, pericardial edema, reduced pigmentation and hematopoietic defects (Amsterdam et al., 2004a; Danilova et al., 2011; Taylor et al., 2012; Zhang et al., 2014). Similar phenotypes were observed in zebrafish embryos in which *rps19* or *rpl11* were knocked down using morpholinos; in addition, some morphants had missing eyes, abnormal positioning of the heart and pancreas, and increased fin size (Chakraborty et al., 2009; Danilova et al., 2008b; Uechi et al., 2008). Adult zebrafish heterozygous for RP mutations have a high frequency of malignant peripheral nerve sheath tumors (Amsterdam et al., 2004b).

In mice, mutations in *Rps19* and *Rps20* cause dark skin, reduced body size and a reduced erythrocyte count (McGowan et al., 2008). RPS6-deficient mice also have hematopoietic defects (Keel et al., 2012). A transgenic mouse carrying an *Rps19* R62W mutation, which is common in DBA, has been recently developed (Devlin et al., 2010). The constitutive expression of the transgene bearing this mutation is developmentally lethal. Its conditional expression results in growth retardation and anemia. Another RPS19-deficient mouse created by transgenic RNA interference also develops symptoms similar to those found in individuals with DBA (Jaako et al., 2011).

**Fig. 1.**
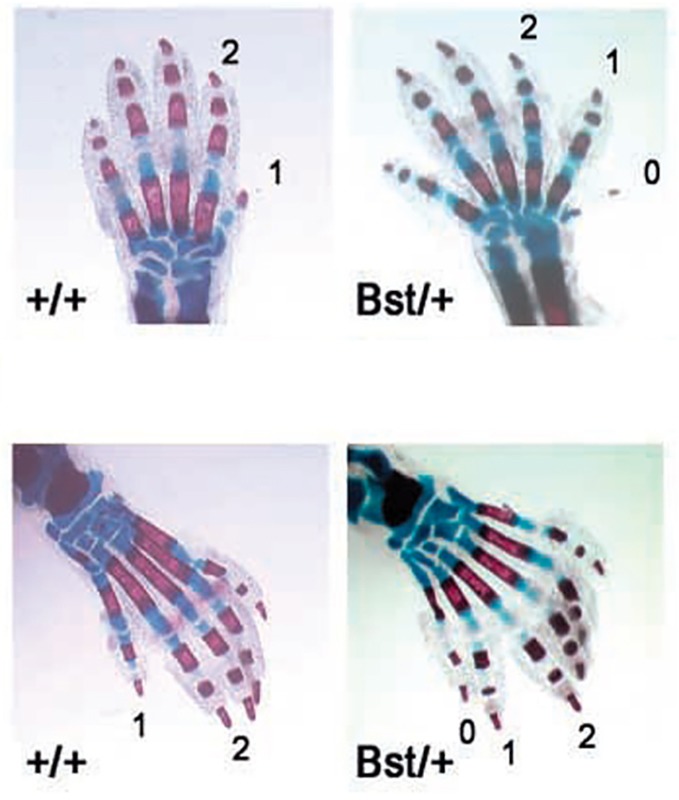
**Duplicated digits and phalanges in mice heterozygous for *Rpl24*.** Skeletal stain of newborn forelimbs (upper) and hindlimbs (lower). Mice heterozygous for a mutation in *Rpl24* (*Bst*/+ phenotype) show preaxial polydactyly (0) and triphalangy of the first digit (1). Figure reproduced with permission (Oliver et al., 2004).

Mutation of the mouse *Rpl24* leads to the Belly Spot and Tail (Bst) phenotype characterized by small size, eye defects, a white ventral spot, white hind feet and various skeletal abnormalities including duplicated digits and phalanges (Fig. 1), which is similar to the anomaly noted in individuals with DBA (Oliver et al., 2004). Mutations in *Rps7* also lead to skeletal malformations, ventral white spotting and eye defects (Watkins-Chow et al., 2013).

Thus, several cross-species features caused by RP deficiency have emerged, which include a smaller size, eye defects, congenital malformations often associated with the duplication or overgrowth of structures derived from the epidermal ectoderm, and varying degrees of anemia.

### Examples of phenotypes of other ribosomopathies

Treacher Collins syndrome (TCS) is caused by mutations in genes involved in rRNA transcription, such as the Treacher Collins-Franceschetti syndrome 1 (*TCOF1*) and *POLR1D* and *POLR1C* genes, which encode subunits of RNA polymerase I and III (PolI/III; Box 1) (Dauwerse et al., 2011; Valdez et al., 2004). TCS is associated with craniofacial deformities such as absent cheekbones (Dixon et al., 2006).

Shwachman Diamond syndrome (SDS) is caused by mutations in the Shwachman-Bodian-Diamond syndrome (*SBDS*) gene, which functions in ribosomal subunit joining (Boocock et al., 2003). SDS is characterized by decreased production of white blood cells, exocrine pancreatic insufficiency, and problems with bone formation and growth.

Dyskeratosis congenita (DC) is caused by mutations in genes that code for the components of small nucleolar ribonucleoprotein complexes, which function in rRNA processing (Ruggero et al., 2003). Individuals with DC have mucocutaneous defects and bone marrow failure.

North American Indian childhood cirrhosis (NAIC) is caused by mutation in *CIRH1A* [cirrhosis, autosomal recessive 1A (cirhin)], which encodes a protein that functions in rRNA processing (Chagnon et al., 2002). The phenotype is restricted to neonatal jaundice that progresses to biliary cirrhosis (Box 1) that at some point requires hepatic transplantation.

These examples illustrate the variability of phenotypes of ribosomopathies. A comprehensive description of these and other ribosomopathies can be found in recent reviews (Armistead and Triggs-Raine, 2014; James et al., 2014). How a general defect can lead to the malfunction of a specific tissue remains a focus of many ongoing studies, which uncovered that some phenotypic consequences of ribosomal stress are caused by p53 activation, as discussed in more detail in the next section.

## Abortive ribosome biogenesis and p53 activation

### Ribosome biogenesis: stages and the role of RPs

**Fig. 2.**
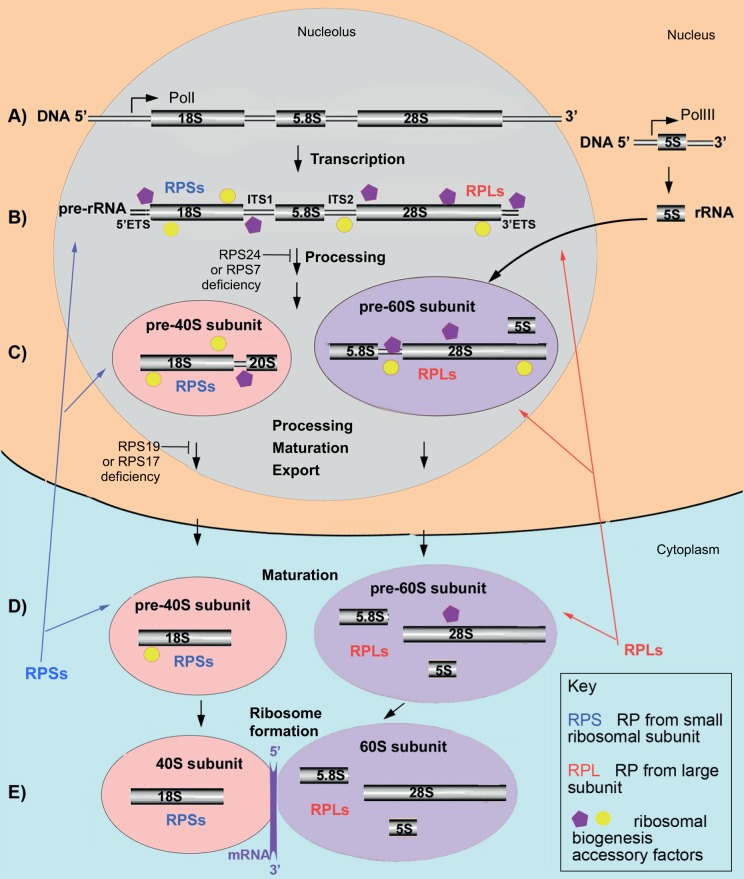
**A simplified schematic of ribosome biogenesis in human cells.** (A) 18S, 5.8S and 28S rRNAs are transcribed by Pol1 in the nucleolus as segments of a long precursor pre-rRNA, which also includes two externally transcribed spacers 5′ETS and 3′ETS and two internally transcribed spacers, ITS1 and ITS2 (B; Box 1). 5S rRNA is transcribed independently by PolIII in the nucleus. (B) Concomitant with transcription, the pre-rRNA assembles with accessory factors and a subset of ribosomal proteins (RPs: RPSs and RPLs). This facilitates the formation of a secondary structure necessary for the correct folding, modification and cleavage of pre-rRNA. (C) After removal of the 5′ETS and cleavage in the ITS1 site, pre-40S (which contains the 20S precursor of 18S rRNA) and pre-60S subunits are formed and continue to mature. 5S rRNA incorporates into pre-60S subunit. Subunits are then exported to the cytoplasm. (D) Once in the cytoplasm, small and large subunits undergo final maturation, which involves the removal of remaining accessory factors and incorporation of missing RPs. (E) A functional ribosome forms after transcribed mRNA binds to the 40S subunit, which triggers association of the 60S subunit with this complex. More than 200 accessory factors, which include helicases, nucleases, small nucleolar RNAs (snoRNAs; Box 1), chaperones and transporters, temporally associate with the maturing ribosomal subunits at various steps. In human cells, pre-rRNA processing is differentially affected by deficiency of various RPs. For example, deficiency of RPS24 or RPS7 prevents formation of the 20S precursor of 18S rRNA, whereas deficiency of RPS19 or RPS17 prevents conversion of the 20S precursor to a mature 18S rRNA.

The eukaryotic ribosome is composed of a small (40S) and a large (60S) subunit (Kressler et al., 2010). The small subunit includes 18S rRNA and 33 RPs; the large subunit includes 5S rRNA, 28S rRNA, 5.8S rRNA and 46 RPs. The genes encoding rRNA (rDNA) are found in multiple copies organized into tandem repeats. 18S, 5.8S and 28S rRNAs are transcribed as a single pre-rRNA transcript by RNA polymerase I in a substructure of the nucleus called the nucleolus. In yeast, the rDNA repeat also encodes the 5S rRNA, which is transcribed in the reverse direction (Henras et al., 2015). In human cells, the 5S rRNA precursor is transcribed from multiple genes in the nucleoplasm by RNA polymerase III (Henras et al., 2015). Then, 5S rRNA migrates to the nucleolus for further processing and incorporation into the pre-60S subunit. The nascent pre-rRNA assembles co-transcriptionally with a subset of RPs and with multiple other factors that facilitate the folding, modification and cleavage of pre-rRNA and the formation of ribosomal subunits (Fig. 2). Pre-rRNA processing can take place co-transcriptionally as well as post-transcriptionally. In yeast, the pre-40S subunit most often is released by cleavage within the internal transcribed spacer 1 (ITS1) before the transcription of the 3′ end of the pre-rRNA is finished. Details of pre-rRNA processing can be found in recent reviews (Henras et al., 2015; Kressler et al., 2010).

Most RPs are strictly required for the pre-rRNA processing. It is thought that their role is to assist the proper folding of the pre-rRNA (Henras et al., 2015). RPs act in a hierarchical order. Approximately half of RPs from the 40S ribosomal subunit, including RPS24 and RPS7, associate early with the 5′ end of the nascent pre-rRNA and are required for the initiation of cleavages at the 5′ external transcribed spacer (5′ETS; Box 1) and ITS1 (Fig. 2). The second set of RPs, which includes RPS19 and RPS17 along with other RPs, is not required for 5′ETS removal but is necessary for ITS1 cleavage. Correspondingly, depletion of RPS24 or RPS7 in yeast and human cells results in the failure of maturation of the 5′ end of 18S rRNA, whereas, in the absence of RPS19 or RPS17, the 3′ end of the 18S rRNA cannot mature (Choesmel et al., 2007, 2008; Flygare et al., 2007; Leger-Silvestre et al., 2005; Robledo et al., 2008). In all cases, pre-40S particles that contain non-cleaved pre-rRNA cannot be exported and accumulate in the nucleus. Similarly, deficiency of most other RPs that are mutated in DBA, both from the small and the large ribosomal subunits, affects pre-rRNA processing in a unique way, leading to the accumulation of different rRNA precursors and disruption of ribosome biogenesis at different steps (Robledo et al., 2008).

### RP deficiency and p53 activation

Ribosomal biogenesis is a highly energy-consuming process that is coupled to cell growth and requires tight regulation. It is controlled by the TOR pathway, which regulates the synthesis of ribosomal components in response to growth factors and nutrient availability (see Box 1 for a glossary of terms) (Kressler et al., 2010). Ribosome biosynthesis requires coordinated activity of three RNA polymerases, PolI, PolII and PolIII. Cellular stress such as hypoxia or DNA damage decreases ribosome biogenesis mostly by inhibition of PolI transcription of rRNA through several mechanisms (Boulon et al., 2010). Shutting down ribosome biogenesis during stress preserves cellular homeostasis and ensures that enough cellular resources can be relocated to a response to stress.

One of the first indications that p53 is involved in controlling the fidelity of ribosome biogenesis came from the finding that a mutation in *Bop1*, which is involved in maturation of rRNAs, leads to p53-dependent cell cycle arrest in mouse cells (Pestov et al., 2001) and that *RPS6*-gene haploinsufficiency activates p53 in mouse embryos and T cells (Panic et al., 2006; Sulic et al., 2005). Subsequent studies have established p53 activation as a general response to RP deficiency (Barlow et al., 2010; Danilova et al., 2008b; Dutt et al., 2011; Fumagalli and Thomas, 2011; McGowan et al., 2008; Taylor et al., 2012). Acquired loss of RPS14 in 5q-myelodysplastic syndrome (5q-MDS; Box 1) is also associated with p53 upregulation (Dutt et al., 2011; Pellagatti et al., 2008).

Several hypotheses to explain how p53 is activated in DBA have been proposed. The nucleolus has been suggested to be a universal stress sensor that is responsible for the maintenance of a low level of p53 in the cell; the nucleolus disrupts and arrests the cell cycle in response to various stresses, including DNA damage, hypoxia, heat shock, nucleoside triphosphate (NTP) depletion, and others (Rubbi and Milner, 2003). However, RP deficiency does not always lead to nucleolar disruption (Fumagalli et al., 2009), suggesting that other mechanisms sense ribosomal stress as well.

**Fig. 3.**
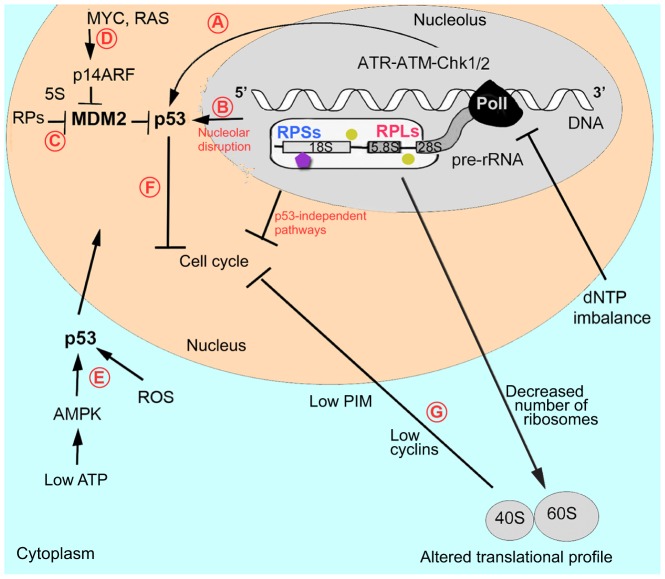
**Defects in ribosomal biogenesis activate p53 and other stress-response mechanisms.** A schematic showing pre-rRNA transcription, and assembly of accessory factors and RPs on the nascent pre-rRNA. (A) Recent studies have suggested that problems with pre-rRNA processing can affect DNA transcription, leading to the activation of ATR-ATM-Chk1/2 signaling (which is responsible for the replication-stress and DNA-damage checkpoints) and p53 upregulation. In addition, deoxynucleoside triphosphate (dNTP) imbalance caused by RP deficiency might interfere with transcription and replication and contribute to ATR-ATM activation. (B) Problems with pre-RNA processing compromise ribosome biogenesis and lead to nucleolar disruption. The nucleolus is involved in maintaining low p53 levels by exporting it for degradation. Various stressors disrupt nucleolar organization, compromising p53 export and leading to p53 accumulation in the nucleus. Nucleolar disruption might also lead to the release of factors that activate p53 or cause cell cycle arrest by p53-independent mechanisms. (C) An alternative pathway of p53 activation is through free RPs that, in complex with 5S RNA, bind the p53 negative regulator MDM2, releasing p53 from its control. (D) Upregulation of MYC and RAS pro-survival factors in DBA patients and in animal models suggests that they might activate p14ARF, which, in complex with 5S RNA, also negatively regulates MDM2. Hypothetically, additional not-yet-identified nucleolar factors might also negatively interact with MDM2 and contribute to p53 upregulation. (E) p53 might also be activated by secondary changes in RP-deficient cells, such as increased levels of ROS or decreased levels of ATP, which activates AMPK, which, in turn, activates p53. p53 then translocates to the nucleus. (F) p53 activation leads to cell cycle arrest and to the induction of downstream pathways ranging from cellular repair to apoptotic mechanisms. (G) A p53-independent response might also originate from the cytoplasm owing to a decreased number and altered activity of ribosomes, which also can lead to cell cycle arrest. For example, decreased levels of cyclins or PIM1 caused by RP deficiency might inhibit cell cycle progression. Abbreviations: AMPK, AMP-activated protein kinase; ATM, ataxia telangiectasia mutated; ATR, ataxia telangiectasia and Rad3 related; Chk1/2, checkpoint kinase 1/2; MDM2, MDM2 oncogene, E3 ubiquitin protein ligase; MYC, avian myelocytomatosis viral oncogene homolog; p14ARF, alternate reading frame protein product of the CDKN2A, cyclin-dependent kinase inhibitor 2A; PIM, pim-1 oncogene; PolI, RNA polymerase I; RAS, rat sarcoma viral oncogene homolog; ROS, reactive oxygen species; 5S, rRNA. See Fig. 2 for a key.

One way in which altered ribosome biogenesis might affect p53 is through RPs that are not incorporated into ribosomes (Fig. 3). Several RPs have been shown to bind Mdm2 (mouse double minute 2 homolog, which is a negative regulator of p53) and to inhibit its binding to p53, leading to p53 stabilization and to cell cycle arrest (Zhang and Lu, 2009). Attaining the right balance between the synthesis of rRNA and RPs seems to be important; when rRNA synthesis is decreased, RPs are no longer used for ribosome building and can stabilize p53 (Donati et al., 2011). Thus, p53 stabilization by RPs might be a general mechanism involved in the response to various stresses.

RPL5 and RPL11 have been proposed to be the essential players in the regulation of p53 by RPs (Macias et al., 2010). Together with 5S rRNA they form the Mdm2 regulatory complex (Sloan et al., 2013). They are protected from the proteasomal degradation that other RPs undergo when PolI is inhibited (Bursac et al., 2012). This raises the question of how cells respond to a deficiency of RPL5 or RPL11. In primary human lung fibroblasts and in murine embryonic stem cell lines, their depletion reportedly does not induce the p53 pathway (Teng et al., 2013; Singh et al., 2014). However, Rpl11-deficient zebrafish do have an upregulated p53 pathway (Chakraborty et al., 2009; Danilova et al., 2011). These contradictory results flag the need for further investigation into the mode of action of these RPs in p53 regulation.

Besides the RPs, Mdm2 has over a hundred binding partners and some of them might be involved in response to ribosomal stress (James et al., 2014). One of them, p14ARF, an alternate reading frame protein product of cyclin-dependent kinase inhibitor 2A (*CDKN2A*), is an Mdm2 inhibitor and is upregulated in response to oncogenic signaling. An increase in such signaling has been reported in hematopoietic progenitors derived from DBA patients (Gazda et al., 2006a) and in Rpl11-deficient zebrafish (Danilova et al., 2011) (Fig. 3).

Some data suggest that ataxia telangiectasia and Rad3 related (ATR) and ataxia telangiectasia mutated (ATM) kinases, which are responsible for the replication stress and DNA-damage checkpoints, might contribute to p53 upregulation in situations where rRNA synthesis is compromised (Fig. 3). The ATR-Chk1 axis was implicated in cell cycle arrest induced by inhibition of rRNA synthesis using actinomycin D (Ma and Pederson, 2013). Another study reported upregulation of the ATR-ATM-Chk1-p53 pathway in RPS19-deficient human cells and in zebrafish models of DBA (Danilova et al., 2014). The exact mechanism of ATR induction in these studies was not explored. However, some researchers have hypothesized that the obstruction of pre-rRNA processing might interfere with transcription and, ultimately, with DNA replication (Bermejo et al., 2012). This hypothesis is based on the fact that pre-rRNA processing starts before its transcription is finished (Osheim et al., 2004; Schneider et al., 2007). Pre-mRNA splicing also happens co-transcriptionally and splicing defects increase the formation of DNA double-strand breaks (Li and Manley, 2005).

An additional source of p53 activation in RP-deficient cells might arise from the altered nucleotide metabolism (Fig. 3). In RP-deficient zebrafish, the level of adenosine triphosphate (ATP), which is a source of energy, is decreased, whereas that of deoxythymidine triphosphate (dTTP), which is a building block of DNA, is increased (Danilova et al., 2014). Decreased ATP leads to activation of the energy sensor AMP-activated protein kinase (AMPK), which, in turn, activates p53 (Hardie et al., 2012). The disproportional increase or decrease in one of deoxyribonucleotide triphosphates can interfere with DNA synthesis and result in p53 upregulation (Austin et al., 2012; Sanchez et al., 2012).

RP deficiency in a fetal environment, in addition to p53 protein stabilization, leads to the transcriptional upregulation of p53 (Chakraborty et al., 2009; Danilova et al., 2008b) possibly because of the upregulation of certain growth factors such as Myc (Danilova et al., 2011).

Reactive oxygen species (ROS) can also contribute to p53 stabilization in RP-deficient cells (Heijnen et al., 2014). The insufficient production of hemoglobin and the accumulation of the excess of heme might contribute to oxidative stress in erythroid progenitors (Ellis, 2014).

Various hypotheses of p53 activation are not mutually exclusive. In fact, several mechanisms might function in parallel to induce p53 to guard ribosome biogenesis. The contribution of each mechanism could differ between species, tissues, and with the particular RP involved.

### p53 activation in other ribosomopathies

p53 activation occurs in other ribosomopathies as well. The craniofacial defects in TCS are mediated by p53 upregulation (Jones et al., 2008). p53 protein overexpression has been found in bone marrow biopsies of patients with SDS (Elghetany and Alter, 2002). In zebrafish and mouse models of DC, p53 is upregulated (Pereboom et al., 2011; Zhang et al., 2012; Gu et al., 2008). Knockdown of the zebrafish homolog of the gene responsible for NAIC results in p53 activation (Wilkins et al., 2013).

## p53-independent responses to ribosomal stress

Recent studies of transcriptional responses to RP deficiency induced in p53-negative human cell lines revealed changes in the expression of genes involved in metabolism, proliferation, apoptosis and cell redox homeostasis (Aspesi et al., 2014). Studies of murine embryonic stem cells haploinsufficient for *Rps19* and *Rpl5* indicate the presence of p53-independent cell-cycle and erythroid-differentiation defects (Singh et al., 2014). Zebrafish studies also point to both p53-dependent and -independent responses to RP deficiency (Danilova et al., 2008b; Torihara et al., 2011). Yeast, an organism that lacks the p53-Mdm2 pathway, responds to the inhibition of ribosome biogenesis by cell cycle arrest, which involves a yeast functional equivalent of the human tumor suppressor pRb (protein retinoblastoma) (Bernstein et al., 2007). Some p53-independent responses to ribosomal stress are outlined below.

### Reduced ribosome number or activity

Reduced ribosome activity might contribute to DBA phenotypes. Slowed proliferation of human RPL11- or RPL5-deficient lung fibroblasts was attributed to reduced ribosome content and translational capacity that suppressed the accumulation of cyclins and thus cell cycle progression (Teng et al., 2013). Although reduced ribosome number and activity might still be sufficient for survival, they might be not adequate to translate some mRNAs that have stringent conditions for initiation of translation (Ludwig et al., 2014). Moreover, ribosomal stress affects translation of internal ribosome entry site (IRES)-containing RNAs (Armistead and Triggs-Raine, 2014; Horos et al., 2012) (Box 1). Thus, ribosomal stress might lead to changes in the spectrum of translated mRNAs, which could differ between tissues.

### Ribosomes as a platform for kinase signaling

Ribosomes were suggested to be a platform for signaling molecules after the discovery that RAC1, a receptor for protein kinase C, is a constituent of the ribosome (Nilsson et al., 2004). The PIM1 proto-oncogene serine/threonine kinase is also associated with ribosomes (Chiocchetti et al., 2005). In human erythroid cell lines, RP deficiency and other types of ribosomal stress such as inhibition of rRNA production at various steps with actinomycin, camptothecin or cisplatinum cause a decrease of PIM1 levels, leading to inhibition of the cell cycle (Iadevaia et al., 2010). A pathway conserved from yeast to mammals involves the regulation of growth-related processes via the interaction between ribosomes and TORC2 (see Box 1 for a glossary of terms), and ribosomal defects can inhibit TORC2 (Zinzalla et al., 2011). These mechanisms might contribute to the phenotypes of RP-deficient cells.

### RPs as regulators

In addition to their structural role in the ribosome, RPs are involved in other cellular processes. Several RPs modulate the activity of regulatory proteins such as NF-κB, p53, p21, Myc and nuclear receptors, whereas others regulate translation by binding to untranslated regions of mRNAs (Warner and McIntosh, 2009). Some RPs act as a regulatory component of the ribosome to confer transcript-specific translational control. For example, translation of vesicular stomatitis virus mRNAs depends on RPL40 (Lee et al., 2013). This RP is also required for translation of some cellular mRNAs, including those involved in the stress response (Lee et al., 2013). RPS25 facilitates interactions of the ribosome with viral IRES elements (Landry et al., 2009). In *Rpl38*-mutant mouse embryos, global translation is unchanged but the translation of a subset of homeobox mRNAs is perturbed (Kondrashov et al., 2011). An imbalance in RP regulatory functions might contribute to the pathophysiology of DBA. For example, increased frequency of physical malformations in individuals with mutations in *RPL5* and *RPL11* (Gazda et al., 2008) might be related to impaired regulatory functions of these RPs.

## Pathological changes in RP-deficient cells

Both p53-positive and p53-negative animal cells respond to RP deficiency by global homeostatic changes that serve to stall proliferation and to create conditions for cellular repair, senescence or apoptosis depending on the cell type and the intensity of the stress signal. Here, we outline the most significant changes that have been observed in RP-deficient cells, both p53-dependent or -independent. A better understanding of these changes might help to find new treatments for DBA and other ribosomopathies.

### Cell cycle arrest and apoptosis

An increase in the expression of genes responsible for cell cycle arrest has been reported in various DBA models (Barkic et al., 2009; Danilova et al., 2008b; Miyake et al., 2008). Slower cell proliferation might promote repair and improve survival (Barkic et al., 2009). However, when repair is not possible, cells activate apoptosis. Increased apoptosis has been reported in animal and cellular models of DBA, as well as in cells from DBA patients (Avondo et al., 2009; Danilova et al., 2011, 2008b; Gazda et al., 2006a; Miyake et al., 2008; Perdahl et al., 1994). p53-negative cells also respond to RP deficiency by the inhibition of proliferation and by the upregulation of pro-apoptotic genes (Aspesi et al., 2014).

### Metabolism

Rpl11-deficient zebrafish and RPS19-deficient mouse fetal liver cells downregulate genes that encode glycolytic enzymes and upregulate genes involved in aerobic respiration (Danilova et al., 2011). This is consistent with a known effect of p53 activation (Matoba et al., 2006). Glycolysis provides not only ATP but also intermediates for the biosynthesis of carbohydrates, proteins, lipids and nucleic acids, and is increased in normal cells during proliferation and in tumors, which is known as the Warburg effect (Lunt and Vander Heiden, 2011). Suppressed glycolysis in RP-deficient cells means that lower levels of intermediates are available for the biosynthesis of organic macromolecules. Moreover, in Rpl11-deficient zebrafish, the expression of genes encoding enzymes that are involved in the biosynthesis of lipids and proteins is downregulated, whereas expression of genes involved in catabolism is upregulated (Danilova et al., 2011). Changes in the expression of genes involved in metabolism have also been found in p53-negative RP-deficient cells (Aspesi et al., 2014).

Autophagy is a mechanism used by normal cells, but especially by stressed cells, to replenish their energy and biosynthetic intermediates. Consistently, disrupted ribosome biogenesis leads to the activation of autophagy both in human cells and in zebrafish embryos (Heijnen et al., 2014; Boglev et al., 2013).

Expression of genes encoding enzymes involved in ROS detoxification, such as superoxide dismutase 2, is decreased both in *Rpl11* zebrafish mutants and in p53-negative RPS19-deficient human cell lines (Aspesi et al., 2014; Danilova et al., 2011). These results indicate that RP-depleted cells are predisposed to oxidative stress.

A common metabolic change in DBA is the upregulation of ADA (Fargo et al., 2013). Overexpression of ADA causes ATP depletion (Chen and Mitchell, 1994), which was also found in RP-deficient zebrafish (Danilova et al., 2014). ATP shortage leads to the activation of AMPK, which inhibits biosynthesis and translation (Hardie et al., 2012). Translation is decreased in RP-deficient cells (Cmejlova et al., 2006; Gazda et al., 2006a; Signer et al., 2014) despite the overstimulation of mTOR and S6 kinase that is found in these cells (Heijnen et al., 2014; Payne et al., 2012).

Another important metabolic change in RP-deficient cells is the dysregulation of the insulin pathway. Recent data suggest that RP deficiency leads to the inhibition of this pathway through a mechanism that is reminiscent of insulin resistance (Heijnen et al., 2014). Increased glucose levels and the upregulation of pre-proinsulin are also observed in zebrafish heterozygous for the *rpl11* mutation (Danilova et al., 2011). Known processes contributing to the development of insulin resistance include p53 activation, increased ROS and increased production of proinflammatory cytokines (Minamino et al., 2009).

### Structural changes

Multiple changes in the expression of genes that encode structural proteins have been found in an *rpl11* zebrafish mutant, such as the downregulation of collagens and the membrane components of red blood cells (Danilova et al., 2011). In DBA patients, the composition of the membranes of red blood cells also changes, with the accumulation of non-red blood cell proteins (Pesciotta et al., 2014).

### Activation of the innate immune system

The upregulation of genes involved in interferon and TNFα (tumor necrosis factor alpha) signaling has been reported in red blood cell progenitors and in fibroblasts obtained from DBA patients (Avondo et al., 2009; Gazda et al., 2006a), as well as in Rpl11-deficient zebrafish (Danilova et al., 2011). TNFα signaling has been found to contribute to the hematopoietic failure observed in RPS19-deficient human hematopoietic progenitors and in Rps19-deficient zebrafish embryos (Bibikova et al., 2014). In RP-deficient zebrafish, the upregulation of several components of the complement system and the downregulation of complement inhibitors have been reported (Danilova et al., 2011; Jia et al., 2013). Notably, monocytes from the bone marrow of DBA, DC and SDS patients have an altered response to bacterial antigens (Matsui et al., 2013).

### Cancer

The increased incidence of cancer in an RP-deficient environment might be attributed to upregulation both of factors that induce apoptosis and factors that promote proliferation, as was reported for zebrafish *rpl11* mutants (Danilova et al., 2011). Also, in hematopoietic progenitors from DBA patients, some members of the RAS family are upregulated, whereas the tumor suppressors breast cancer 2, early onset (*BRCA2*) and retinoblastoma 1 (*RB1*) are downregulated (Gazda et al., 2006a). The RP-deficient condition therefore resembles an after-irradiation environment in which both pro-apoptotic and survival factors are upregulated; such an environment confers a dramatic selective advantage to p53-deficient cells that avoid cell cycle arrest and apoptosis (Marusyk et al., 2010). Selection for p53-negative cells takes place at the level of progenitor cells, which are the most sensitive to stress. Interestingly, in RP-deficient zebrafish tumors, the *tp53* gene is wild type and transcribed, but the p53 protein is not synthesized (MacInnes et al., 2008). The mechanism of the selective suppression of p53 translation in these tumors is currently unknown.

## Failure of erythropoiesis in DBA

Erythroid failure is the most distinctive feature of DBA, with decreased proliferation of erythroid progenitors (Ebert et al., 2005; Flygare et al., 2005) and their increased sensitivity to apoptosis (Perdahl et al., 1994). In RP-deficient erythroid progenitors, p53 is selectively activated (Dutt et al., 2011). Hematopoietic defects appear already at the stage of hematopoietic stem cells (HSCs). Their number in RP-deficient zebrafish is decreased (Danilova et al., 2011) and induced pluripotent stem cells derived from DBA patients exhibit impaired differentiation (Garcon et al., 2013). In this section, we discuss factors that might contribute to erythroid defects in DBA.

### Decreased GATA1 levels

Mutations in the erythroid-specific gene *GATA1* (GATA binding protein 1) leads to a phenotype that is currently classified as DBA (Sankaran et al., 2012). Although individuals with *GATA1* mutations do not have congenital malformations, their erythroid phenotype is very similar to that of DBA-affected individuals with RP mutations. Recent studies demonstrated that RP knockdown in primary human hematopoietic progenitors or in an erythroid cell line leads to decreased *GATA1* translation (Bibikova et al., 2014; Ludwig et al., 2014). A decreased level of Gata1 protein was also found in zebrafish mutant for *rpl11* (Bibikova et al., 2014). Moreover, a global gene expression profiling of erythroid cells from DBA patients showed that the expression of GATA1 transcriptional target genes is downregulated in these cells (Ludwig et al., 2014), which is consistent with decreased GATA1 activity. These data suggest that a decreased level of GATA1 protein underlies many defects of erythroid cells in DBA (Ludwig et al., 2014). In support of this, when GATA1 protein levels were increased in bone marrow mononuclear cells from DBA patients or in primary human hematopoietic cells with reduced levels of RPL11 or RPL5, red blood cell production and cell differentiation was improved (Ludwig et al., 2014). The mechanism of GATA1 suppression in DBA might involve the highly structured 5′ end of *GATA1* mRNA that requires stringent conditions for translation initiation (Ludwig et al., 2014). These findings show that GATA1, although not the only target of translation dysregulation in DBA, is an important factor in mediating the erythroid-specific defect observed in this condition.

### Increased proliferation causes increased demand for ribosomes

It has been suggested that, because the chromatin of erythroid cells becomes condensed and transcriptionally inactive prior to enucleation, the rapidly proliferating immature erythroid cells require very high ribosome synthesis rates in order to produce enough ribosomes to last for the duration of the mature erythrocyte life cycle (Sieff et al., 2010). This hypothesis was confirmed in fetal liver cells of RP-deficient mice, in which cell numbers increased three to fourfold while RNA content increased sixfold, suggesting an accumulation of an excess of ribosomes during early erythropoiesis (Sieff et al., 2010).

In addition, rapid proliferation itself might make cells vulnerable to stressful conditions. It is known that irradiation does the most damage to hematopoietic and epithelial tissues, including the thymus, spleen and small intestine, where cell turnover is the fastest; this sensitivity correlates with the increased induction of p53 in these tissues (Gottlieb et al., 1997; Komarova et al., 1997).

### Erythroid-specific alteration in transcription, splicing or translation

Some data suggest that erythroid progenitors respond to stress by selective changes in transcription, splicing or in the translation of a specific set of genes. The decreased transcription of a crucial erythropoietic factor, MYB (v-myb avian myeloblastosis viral oncogene homolog), has been reported in erythroid progenitors from DBA patients (Gazda et al., 2006a) and in Rpl11-deficient zebrafish (Danilova et al., 2011). Myb protein levels are also reduced in RP-deficient mouse fetal liver cells (Sieff et al., 2010).

Splicing is a process that can be selectively affected under stress (Dutertre et al., 2011). Immature erythroid cells from DBA patients express alternatively spliced, non-functional isoforms of FLVCR1 (feline leukemia virus subgroup C cellular receptor 1), a heme exporter required for erythropoiesis (Rey et al., 2008). These results suggest that FLVCR1 insufficiency contributes to the erythropoietic defects seen in individuals with DBA.

There are indications of increased translational suppression of erythroid-specific genes in RP-deficient organisms in comparison to other genes. In addition to the selective suppression of GATA1 translation discussed above, selective suppression of globin translation has been reported in RP-deficient zebrafish (Zhang et al., 2014). Decreased protein levels of some common factors that are normally highly expressed in differentiating erythroid cells, such as BCL2-associated athanogene (*BAG1*), which encodes a HSP70 co-chaperone, and cold shock domain containing E1, RNA-binding (*CSDE1*), have been reported in mouse RP-deficient erythroblasts and erythroblasts cultured from DBA patients (Horos et al., 2012).

Additional suppression of GATA1 protein levels might be mediated through the upregulation of TNFα in RP-deficient cells (Bibikova et al., 2014).

### Metabolic and structural vulnerability of erythroid cells

In addition, red blood cells have a distinct physiology that might make them selectively vulnerable to alterations in several common pathways. An example is the shift from glycolysis to aerobic respiration, which takes place in all cells after p53 upregulation (Matoba et al., 2006), including in RP-deficient cells (Danilova et al., 2011). Although this shift occurs in all cells of RP-deficient organisms, only erythrocytes rely almost exclusively on glycolysis and they are, therefore, selectively affected by this change.

Many membrane and cytoskeleton changes seen in DBA animal models are not specific to erythroid cells but occur in all tissues (Danilova et al., 2011). However, defects originating from these changes might particularly affect erythrocytes because of the especially high requirements for a strong and deformable cell structure to withstand circulation in narrow blood capillaries (Steiner and Gallagher, 2007).

Furthermore, erythroid cells mature in association with macrophages; alterations in membranes of RP-deficient erythroid cells might lead to their increased elimination by macrophages.

## Tissue specificity and variability of defects in ribosomopathies

DBA is characterized by broad phenotypic variability; moreover, some first-degree relatives of DBA-affected individuals with *RPS19* mutations carry an identical mutation but display no observable phenotype (Willig et al., 1999). Congenital malformations are found in ∼40% of DBA patients and they differ between affected individuals. No correlation between the type of mutation and the phenotype has been found among individuals with DBA with *RPS19* mutations (Willig et al., 1999). Similar to DBA, the phenotype of TCS is highly variable and some mutation carriers, parents of patients, are only mildly affected (Dixon and Dixon, 2004; Teber et al., 2004). These findings point to a role for genetic modifiers in the penetrance and severity of both DBA and TSC, and to the importance of a patient's overall genetic background. Below we discuss the origin of specific defects and factors that might cause phenotypic variability in ribosomopathies.

### Role of *ΔNp63* in DBA congenital malformations

**Fig. 4.**
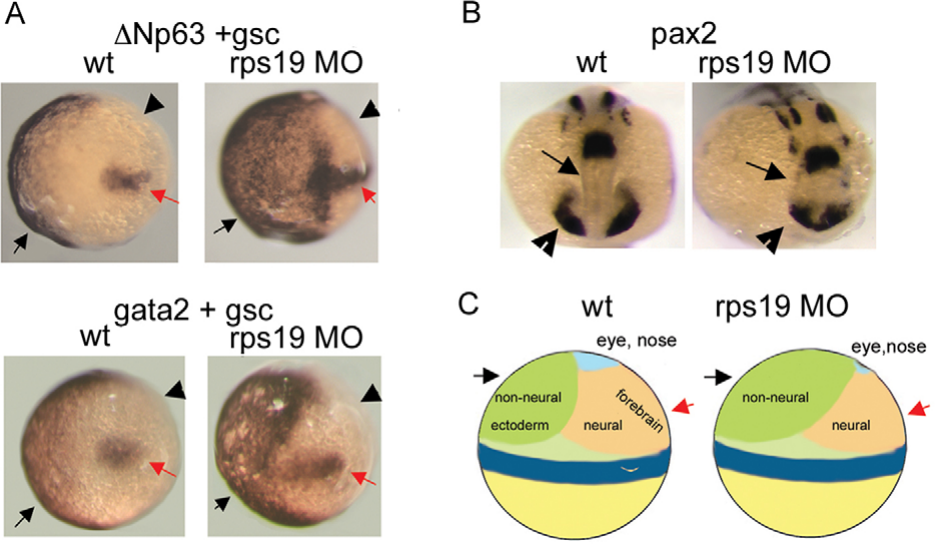
**Origin of developmental defects in RP-deficient zebrafish embryos.** (A) *ΔNp63* expression (black arrow, upper panels) in early zebrafish embryos defines the non-neural ectoderm field and overlaps with a marker of non-neural ectoderm, *gata2* (black arrow, lower panels). In Rps19-deficient zebrafish [in which *rps19* expression has been knocked down with a morpholino oligonucleotide (MO)], this field is expanded (right upper and lower panels). Staining with a probe for goosecoid (*gsc*), necessary for the formation of the dorsoventral axis of the embryo, marks the dorsal side (red arrow). Arrowheads point to the neural field. This is an *in situ* hybridization image at gastrulation, 80% epiboly (Box 1). Dorsal is to the right. wt, wild type. (B) Expression of *pax2*, which has a key role in the development of the CNS, eyes, urogenital tract and kidneys, is altered in Rps19-deficient zebrafish embryos. Arrows and arrowheads point, respectively, to forebrain and eye fields, which are contracted in Rps19-deficient embryos. This is an *in situ* hybridization image at 16 hpf. (C) Schematics showing how expansion of non-neural ectoderm in early zebrafish embryos leads to the contraction of the neural field, especially the area of the forebrain and eye. This research was originally published in *Blood* (Danilova et al., 2008b). ^©^ American Society of Hematology.

Extra digits or phalanges are among the notable congenital defects observed in individuals with DBA (Halperin and Freedman, 1989) and in the *Rpl24* mouse mutant (Oliver et al., 2004) (Fig. 1). As discussed above in the section devoted to the phenotypes of DBA patients and animal models, eye defects are also a common feature. The combination of these defects suggests that they might originate during development from a misbalance between neural and non-neural ectoderm in the early embryo. Development of non-neural ectoderm is controlled by a member of the p53 protein family, ΔNp63*.* Besides its role during development, *ΔNp63* is a p53 target gene and is upregulated in response to p53 activation (Bourdon, 2007). In the gastrulating zebrafish embryos, *ΔNp63* expression is localized to the ventral side of the embryo and marks non-neural ectoderm; its overexpression leads to the expansion of non-neural ectoderm and to the suppression of neural structures (Bakkers et al., 2002). Later in development *ΔNp63* controls the development of limbs and its overactivity can lead to the duplication of limb structures (Mills et al., 1999). In Rps19-deficient zebrafish embryos, the area of *ΔNp63* expression expands to the dorsal side (Danilova et al., 2008b) (Fig. 4A). This corresponds to the expansion of non-neural ectoderm into the neural field, as confirmed by hybridization with *gata2*, a marker of non-neural ectoderm (Fig. 4A). Shrinkage of the neural field affects mostly the forebrain and eyes, as illustrated by the expression of *pax2*, which has a key role in the development of the CNS, eyes, urogenital tract and kidneys (Krauss et al., 1991) (Fig. 4B,C).

It would be interesting to know whether *ΔNp63* upregulation contributes to eye defects and to overgrown wings in *Drosophila* *Minute* mutants, or to the eye defects and the duplication of fingers and phalanges in mice and humans with RP deficiency.

*ΔNp63* is a part of the p53 protein family network, which involves hundreds of proteins that are differentially regulated in various tissues during development (Danilova et al., 2008a). Because both environmental and genetic factors can modulate this network, *ΔNp63* and other members of this network might contribute to phenotypic variation of ribosomopathies. A multitude of other modifiers might theoretically affect cellular response to ribosomal stress, as was recently discussed (Farrar and Dahl, 2011).

### Mechanisms causing dissimilar phenotypes in DBA and TCS

p53 activation contributes to the clinical features of both DBA and TCS. However, these two diseases have completely different phenotypes. In contrast to the bone marrow failure and various tissue defects seen in DBA, TSC defects are restricted to the craniofacial tissues that originate from the neural crest. Most cells in the embryo seem to survive *TCOF1* or *PolI/III* insufficiencies despite their crucial role in ribosome biogenesis. To explain the paradox, it was hypothesized that different progenitor cell populations at defined points during embryonic development have diverse ribosome requirements (James et al., 2014).

Another explanation for this paradox might reside in the spatiotemporal expression patterns of the affected genes. In zebrafish embryos, *tcof1* is expressed at a high level at the one-cell stage, which suggests the maternal origin of its mRNA (Weiner et al., 2012). Moreover, *tcof1* has the highest level of expression at this stage. Therefore, enough protein can be produced in the embryo from the maternal mRNA to pass through the early stages of development. Neural crest cells in zebrafish develop and start to migrate relatively late in development, at ∼15 hours post-fertilization (hpf) through 24 hpf, and form the craniofacial skeleton, the most affected structure in TCS, even later, at ∼72 hpf (Schilling and Kimmel, 1994). At this stage, all zebrafish tissues and organs, including HSCs, are already formed and, therefore, p53 activation can affect only craniofacial tissues. *polr1d*, the zebrafish ortholog of another gene mutated in TCS, is also expressed maternally in zebrafish embryos [Thisse, B., Thisse, C. (2004) Fast Release Clones: A High Throughput Expression Analysis. ZFIN Direct Data Submission (http://zfin.org)].

In contrast to the genes mutated in TCS, only a trace expression of *rpl11* mRNA can be detected at the one-cell stage in zebrafish embryos; it increases from approximately 6 hpf, pointing to the beginning of Rpl11 production (Danilova et al., 2011). p53 upregulation in RP-deficient zebrafish can also be detected from ∼6 hpf (Danilova et al., 2008b), pointing to the onset of problems with ribosome biogenesis at this early time point. Early onset of p53 upregulation in RP-deficient embryos would affect the formation of all tissues and organs. The difference in timing of p53 activation might therefore account for the multitude of defects in DBA versus the relatively restricted phenotype of TCS.

### Specialized ribosomes as drivers of tissue-specific defects

Recent studies suggest that composition of ribosomes can vary between tissues. In yeast, many RPs are duplicated and paralogs have different functional roles (Komili et al., 2007). Based on these data, Komili et al. proposed the existence of many different forms of functionally distinct ribosomes. Recent studies revealed that some animal RPs also have paralogs with specifically tailored functions. Zebrafish Rpl22 and Rpl22L1 play different roles during hematopoiesis (Zhang et al., 2013). Knockdown of *rpl22* blocks the development of T cells, whereas knockdown of *rpl22l1* impairs the development of HSCs. Mice also have Rpl22 and Rpl22L1 paralogs; Rpl22L1 is expressed at a low level in all tissues except pancreas and its expression is regulated by Rpl22 (O'Leary et al., 2013). A recent study found that other RPs and their paralogs are differentially expressed in mouse embryos; the more recently evolved RP paralogs showed a much greater level of tissue-specific expression (Wong et al., 2014).

Not only RPs but other genes involved in ribosome biogenesis also show tissue-specific expression. Cirhin, mutations in which cause childhood cirrhosis (NAIC), is expressed in embryonic mice at the highest levels in the liver, with much weaker expression in other tissues (Chagnon et al., 2002). In zebrafish, cirhin expression is also high in the developing liver (Wilkins et al., 2013).

There might be other ways of creating a different ribosome. For example, RPs, rRNAs and various accessory factors involved in pre-rRNA processing are modified post-transcriptionally and, hypothetically, variations of these processes could also create altered ribosomes. A comprehensive review of these and other mechanisms of the formation of specialized ribosomes have been recently presented (Xue and Barna, 2012).

If ribosomes differ among tissues then mutations in some genes involved in ribosome biogenesis could selectively affect only a subset of ribosomes.

## Current and potential therapeutic approaches to DBA

In spite of recent progress in understanding the pathophysiology of DBA, its treatment is still based on corticosteroids, red blood cell transfusions and HSC transplantation (Vlachos et al., 2014). DBA is a complex disease; it is rather a tree of pathologies. The only way to tackle its cause is with gene therapy and this possibility is being investigated. Replacement of the defective RPs in experimental systems has produced positive results (Flygare et al., 2008; Garcon et al., 2013). This approach holds promise for the future. Until then, the individual consequences of RP deficiency can be targeted.

The most damaging effect of RP deficiency is the upregulation of p53. Direct suppression of p53 in DBA is often considered unacceptable owing to an increased risk of cancer. However, this approach might still be feasible, as illustrated by the effects of cenersen in 5q-MDS. Cenersen is a 20-mer antisense oligonucleotide complementary to *TP53* exon 10; it suppresses p53 expression and restores erythropoiesis in 5q-MDS (Caceres et al., 2013). Zebrafish data suggest that corticosteroids might act, in part, by decreasing the expression of p53 (Danilova et al., 2011).

An indirect way of suppressing p53 activation is suggested by zebrafish studies. Treatment of RP-deficient zebrafish with a mixture of nucleosides decreased the levels of p53, improved hematopoiesis and diminished developmental defects (Danilova et al., 2014). The efficiency of nucleoside treatment can be attributed to the fact that stress changes metabolism. Non-stressed cells use *de novo* nucleotide synthesis to make their DNA; therefore, very few nucleosides from food end up in the DNA of healthy people. Because stressed cells start to use salvage pathways (Austin et al., 2012), nucleoside supplementation improves outcomes in physiologically stressed patients (Hess and Greenberg, 2012). Nucleoside treatment in general decreases replication stress and DNA damage (Bester et al., 2011). Nucleosides in infant formulas have a positive effect on development (Singhal et al., 2010). Nucleoside mixtures are now available as over-the-counter supplements. Their application might benefit DBA patients during the acute phase of the disease.

Inflammation is another damaging result of RP deficiency. Anti-inflammatory properties of lenalidomide, an immunomodulatory agent used to treat several types of cancer, might be responsible for its positive effects in RP-deficient models (Keel et al., 2012; Narla et al., 2011). The TNFα inhibitor etanercept also improves the condition of RP-deficient zebrafish (Bibikova et al., 2014). The upregulation of the complement system in zebrafish DBA models (Danilova et al., 2011; Jia et al., 2013) suggests that complement inhibitors might also merit investigation as a potential treatment for DBA. The careful consideration of the timing of treatments that target p53 or inflammation will still be necessary because these pathways usually antagonize each other (Ak and Levine, 2010).

An important metabolic change in RP-deficient cells that deserves extra attention is the dysregulation of the insulin pathway. Individuals with DBA might have a condition similar to that of pre-diabetes, which could be targeted by the corresponding drugs.

Another important metabolic change, the decrease in translation, has been recently targeted by L-leucine treatment in a DBA patient (Pospisilova et al., 2007). The treatment resulted in an increase in reticulocyte count and hemoglobin levels. Leucine treatment stimulates mTOR and improves anemia in a mouse model of DBA (Jaako et al., 2012). It also decreases anemia and developmental defects in RP-deficient zebrafish embryos and increases proliferation of Rps19- and Rps14-deficient erythroid progenitors (Payne et al., 2012). It is not known, however, what the effect of mTOR overactivation, especially for prolonged periods of time, might be. Several ongoing clinical trials will determine the safety of this treatment in individuals with DBA.

Conflicting signaling seems to be a characteristic of RP-deficient cells, such as the activation of p53 and inflammation. With many modifiers involved, pathways leading from RP deficiency to erythroid defects in one patient might not be exactly the same as in another patient. Moreover, these pathways might differ in the same patient between the acute and remission phases of the disease. Therefore, individualized therapy is likely to be necessary for the successful treatment of DBA.

## Conclusion

A tremendous amount of knowledge about DBA has been accumulated since the first discovery of RP mutation in DBA. DBA studies have stimulated the field of ribosome biogenesis, resulting in a better understanding of the mechanisms of pre-rRNA processing and of ribosome subunit formation. The roles of the p53 and p53-independent pathways in DBA have also become better defined. Multiple diseases have been found to originate from defects in ribosome biogenesis. However, treatment options lag behind this newfound knowledge. The reason for this might lie in the complexity of ribosomopathies, for which a multitude of pathways are involved. Ribosomopathy is a different disease in every patient owing to the effects of genetic background and environment. The factors that modulate an individual response to deficiency of RP or other factors involved in ribosome biogenesis are still not well understood. As such, we need a better understanding of the pathways downstream of p53, as well as the mechanisms of the p53-independent factors that influence the etiology of these diseases.
